# Association between Hypoxia and Perinatal Arterial Ischemic Stroke: A Meta-Analysis

**DOI:** 10.1371/journal.pone.0090106

**Published:** 2014-02-28

**Authors:** Lili Luo, Dapeng Chen, Yi Qu, Jinlin Wu, Xihong Li, Dezhi Mu

**Affiliations:** 1 Department of Pediatrics, West China Second University Hospital, Sichuan University, Sichuan, China; 2 Key Laboratory of Obstetric & Gynecologic and Pediatric Diseases and Birth Defects of Ministry of Education, Sichuan University, Sichuan, China; 3 Department of Emergency, West China Second University Hospital, Sichuan University, Sichuan, China.; University of Birmingham, United Kingdom

## Abstract

**Background:**

Perinatal arterial ischemic stroke (AIS) occurs in an estimated 17 to 93 per 100000 live births, yet the etiology is poorly understood. Although investigators have implicated hypoxia as a potential cause of AIS, the role of hypoxia in AIS remains controversial. The aim of this study was to estimate the association between perinatal hypoxia factors and perinatal arterial ischemic stroke through a meta-analysis of published observational studies.

**Patients and methods:**

A systematic search of electronically available studies published through July 2013 was conducted. Publication bias and heterogeneity across studies were evaluated and summary odds ratios (ORs) and 95% confidence intervals (CIs) were calculated with fixed-effects or random-effects models.

**Results:**

A total of 8 studies describing the association between perinatal hypoxia factors and neonatal arterial ischemic stroke (AIS) met inclusion criteria, and 550 newborns with AIS were enrolled. The associations were found for AIS: preeclampsia (OR 2.14; 95% CI, 1.25 to 3.66), ventouse delivery (OR 2.23; 95% CI, 1.26 to 3.97), fetal heart rate abnormalities (OR 6.30; 95% CI, 3.84 to 10.34), reduced fetal movement (OR 5.35; 95% CI, 2.17 to 13.23), meconium-stained liquor (OR 3.05; 95% CI, 2.02 to 4.60), low Apgar score (OR 5.77; 95% CI, 1.66 to 20.04) and resuscitation at birth (OR 4.59; 95% CI, 3.23 to 6.52). Our data did not show any significant change of the mean risk estimate for oxytocin induction (OR 1.33; 95% CI, 0.84 to 2.11) and low arterial umbilical cord ph (OR 4.63; 95% CI 2.14 to 9.98).

**Conclusions:**

There is a significant association between perinatal hypoxia factors and AIS. The result indicates that perinatal hypoxia maybe one of causes of AIS. Large scale prospective clinical studies are still warranted.

## Introduction

Neonatal stroke is classified as either ischemic or hemorrhagic stroke, and ischemic stroke is further divided into arterial ischemic stroke (AIS) and cerebral sinovenous thrombosis (CSVT) [1]. Perinatal ischemic stroke (PAIS) is defined as a group of heterogeneous conditions in which there is a focal disruption of cerebral blood flow secondary to arterial or venous thrombosis or embolization [2], which occurred from birth up until 28 days postnatal [3].

The prevalence of PAIS has not been clearly determined. Lynch reported an estimated incidence of 71 in 1600 to 5000 births [4]. Laugesaar reported the incidence rate of neonatal stroke in Estonia was 63 per 100 000 live births in 2007 [5]. Lee found that AIS was diagnosed in 20 per 100 000 live births [6]. Estimate incidence rate ranges from 17 to 93 per 100 000 live births [2], [7]–[9]. Possibly due to more widespread use of sophisticated neuro-imaging techniques, recent studies show that the incidence of stroke was significantly higher than before. Meanwhile, a case series of neonates with AIS suggests that newborn boys are at higher risk of ischemic stroke than girls [10], [11], with increased frequency in black children [12]. The incidence of neonatal stroke is higher than old children [5], [13].

Perinatal arterial ischemic stroke is a main cause of cerebral palsy and other neurologic disabilities, thus making it a clinically relevant type of brain injury, yet the etiology is poorly understood.

The pathogenesis of AIS is complex and multifactorial. Risk factors may be related to both maternal and placental problems as well as fetal and neonatal disorders, such as preeclampsia, chorioamnionitis, congenital heart malformations, hemolytic anemias, thrombophilic abnormalities, heart rate abnormalities, resuscitation at birth, low Apgar score, and abnormal cord ph [6], [8], [14]. Investigators have implicated hypoxia as a potential cause of AIS [15]. In a series of 250 newborns examined in the 1980s and 1990s, 35% of strokes occurred in the context of perinatal asphyxia [16]. Some term newborns were found with hypoxic-ischemic encephalopathy, particularly in cases of arterial infarction, (stroke). In a prospective cohort study of 124 term newborns with hypoxia-ischemia encephalopathy, 6 neonates had arterial stroke identified by neuroimaging [17]. Although there was an increased incidence of hypoxia factors in the stroke group, some studies reported there was no significant difference between cases and controls in those diagnosed with birth asphyxia or with meconium-stained liquor [9], [18]. Although a relatively large number of potential risk factors have been implicated in the etiology of AIS, some of the information published to date is conflicting.

The objective of this meta-analysis was to determine the impact of clinical risk factors or markers for hypoxia in neonates with arterial stroke. Our hypothesis is that clinical risk factors for hypoxia differ between newborns with stroke compared to normal controls.

## Methods and Patients

### Study Inclusion Criteria and Exclusion Criteria

Studies included should be published, regardless of its design, publication language or date. Neonatal arterial ischemic stroke is generally defined as a cerebrovascular event occurring from birth to 28 days postnatal with radiological evidence of focal arterial infarction [3], [5]. Published studies of arterial ischemic stroke in infants less than 28 days of age were evaluated for inclusion. We only included studies comparing risk factors between patients with AIS and control subjects. Studies with titles and abstracts discussing risk factors about AIS were further examined, and those where stroke cases were objectively confirmed by suitable imaging methods were included, the suitable imaging methods referred to ischemic lesions revealed by neuro-imaging (CT and/or MR scan). In addition, the included studies have reported the country of origin, study design, ethnicity, and numbers of patients/control subjects. Case reports and case series/studies lacking these controls were excluded, as were cases of cerebral sinovenous thrombosis or hemorrhagic stroke.

### Types of Participants

AIS neonates had to be with neurological clinical symptoms during the 28 first days of life, ischemic lesions in an arterial distribution revealed by suitable imaging methods. Neonates with infection of the central nervous system, brain malformations, venous infarction, cystic periventricular Leukomalaeia (PVL), or sinovenous thrombosis were excluded.

The controls were randomly selected and were individually matched to the infants with PAIS for age and gestational age from the birth registry database or the same birth cohort. Those of a contemporaneous dataset from the general population selected as controls were also included.

### Search Strategy and Identification of Studies

Two investigators (Lili Luo and Dapeng Chen) searched case-control or cohort studies published electronically and listed in database PubMed, EMBASE, Web of Science and Cochrane Library from initial to July 2013. The search terms were perinatal stroke, perinatal and neonatal ischemic stroke, neonatal arterial ischemic stroke, fetal stroke, and presumed prenatal or perinatal arterial ischemic stroke; prepartum complications, intrapartum complications and risk factor. Search consisted of a combination of one term from each group. Potential related studies about the association between hypoxia factors and AIS were first identified by screening the titles and abstracts of candidate articles, full manuscripts were then obtained. Further criteria for inclusion were then applied manually. Furthermore, a manual search was carried out from references cited in these articles. Otherwise we excluded studies with five or fewer cases, because of unreliability.

### Quality Assessment

We used the Newcastle-Ottawa Quality Assessment Scale to assess the quality of both cohort studies and case-control studies [19]. This scale uses an eight-step process that allows for assessment of patient population and selection, study comparability, follow-up, and outcome of interest. Interpretation of the scale is performed by awarding points, (or “stars”), for high-quality elements. Stars are then added up and used to compare study quality in a quantitative manner, as recommended by the Cochrane Non-Randomized Studies Methods Working Group. Studies with 5 or more stars are defined as high quality studies and were included. Quality assessment was independently performed by two investigators (Lili Luo and Dapeng Chen). Data was then independently compiled by two investigators (Lili Luo and Dapeng Chen) after data extraction and assessment. Any areas of discrepancy will be presented in the discussion.

### Data Extraction

For data collection, two investigators used a form of data extraction, independently. Any areas of debate will be presented in the discussion. Studies were included in the meta-analysis only if approved by both investigators. First, we scrutinized in detail the literature on neonatal AIS to identify all possible risk factors which might cause hypoxia. We restricted our analysis to those for which an outcome was assessed by at least 5 cases, because of unreliability. During data extraction we recorded characteristics of the studies, including publication time, study design, country, sample size, gestational week, odds ratio, and 95% confidence intervals (CIs). We have included the studies with the largest number of participants if participant overlap between studies occurred. If original odds ratio and its 95% CI was not reported, the original number of participants was determined in order to calculate the odds ratio.

### Statistical Analysis

We reviewed the possible hypoxia-related risk factors for AIS, included prepartum complications, intrapartum complications and infant characteristics. Among these factors exclusion criteria were: (1) patients less than 5 cases because of the uncertainty; (2) inconsistency of the diagnostic criteria. The association between risk factors and AIS was expressed as odds ratio (OR).

All statistical analysis was performed using Stata 12.0. The pooled OR and its 95% confidence intervals (CIs) were calculated using either the Mantel–Haenszel formula (fixed-effect model) or the Dersimonian–Laird formula (random-effect model). A fixed-effect model was used when heterogeneity was not detected (P>0.10); otherwise, a random-effect model was used. For quantitative evaluation, OR was used to estimate the impact of risk factors on incidence of AIS. OR, variance, 95% CI, log (OR) and se (log (OR)) for each study were extracted or calculated by Stata 12.0 based on the published studies. The weight for each study is the inverse of study variance and variability between studies. A significant two-way P value for comparison was defined as P<0.05. Results are described using forest plots, where a single square represents an individual study's OR estimate. The pooled OR is symbolized by a solid diamond at the bottom of the forest plot and the width of the square represents the 95% CI of the OR. The size of the square represents the weight that the corresponding study exerts in the meta-analysis.

### Assessment of heterogeneity and publication bias

Statistical heterogeneity between studies was examined using both the Q statistic (significant at P<0.1) and the I^2^ value. I^2^>50% were considered to represent significant heterogeneity. Assessment of publication bias was performed for each of the pooled study groups using the Beggar's bias indicator test. The analysis of heterogeneity and publication bias was carried out using the statistical software Stata version 12.0.

## Results

### Search Results and Characteristics of Included Studies

**Figure 1:**
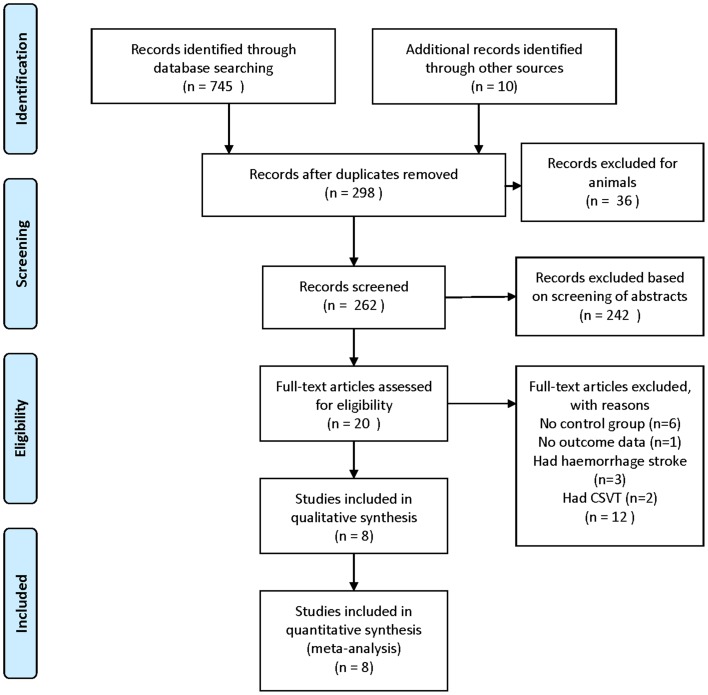
Flow chart showing the results of the search strategy.

We identified 298 publications by our search strategy, except for the duplicate articles. Initial evaluation of abstracts identified 20 related articles, seven case-control studies and one cohort study with 550 patients were selected for analyses. Figure 1 is the flow diagram depicting the reasons for exclusion of the identified studies. The study dates ranged from 1987 to 2010. Table 1 summarizes the characteristics of the studies included in the meta-analysis.

**Table 1:** Characteristic of the included studies.

Study	Years	Country	Study type	Study design	Case number	Controls number	Neonate	Birth weight	Imaging confirm	Risk of bias
Benders 2007 [20]	1990–2005	US	retrospective	case-control	31	93	preterm	Cases:1599±633 Controls:1607±720	MRI	different detecting method, only preterm neonates
Chabrier 2010 [21]	2003–2006	France	prospective	cohort study	100	100	both	NR	CT/MRI	different detecting method, no description of those lost to follow up
Darmency-Stamboul 2012 [22]	2000–2007	France	retrospective	case-control	32	96	term	NR	CT/MRI	different detecting method, only term neonates
Estan 1997 [8]	1987–1993	UK	retrospective	case-control	12	24	term	Cases:3496(2541–4460) Controls:3673 (2748–4707)	CT	different detecting method, only term neonates
Harteman 2012 [14]	2000–2010	Utrecht	retrospective	case-control	52	156	term	Cases:3420(2145–5230) Controls:3520(2155–4925)	MRI	different detecting method, only term neonates
Lee 2005 [6]	1997–2002	US	retrospective	nested case-control	37	111	both	Cases:3127±852 Controls:3203±923	CT/MRI	different detecting method
Wu 2004 [9]	1991–1998	US	retrospective	nested case-control	38	218	term	NR	CT/MRI	different detecting method, only term neonates
Kirton 2011 [23]	2003–2007	IPSS	prospective	case-control	248	population	both	NR	CT/MRI	different detecting method

NR: not reported. IPSS: Europe, Canada, US, South America, Asia, Australia; NR: not reported.

### Selection of Risk factors

After scrutinizing the literature, we yielded 18 possible risk factors (Table 2). Among these factors, forceps delivery, vaginal blood loss, abruption placenta, and infant with congenital heart disease were excluded because of less than 5 cases. We did not include emergency caesarean section, premature rupture of membranes, prolonged second stage of labour and cord abnormality owing to the inconsistency of the diagnostic criteria. Breech delivery was excluded for this factor might or might not lead to hypoxia. Finally, in this meta-analysis, preeclampsia, ventouse delivery, oxytocin induction, fetal heart rate abnormalities, reduced fetal movement, meconium-stained liquor, low Apgar score, abnormal arterial umbilical cord ph and resuscitation at birth were included.

**Table 2:** Potential Hypoxia-related Risk Factors for AIS.

Complications	Numbers of studies	Cases of AIS
Prepartum complications		
Preeclampsia	4	27
Reduced fetal movement	2	16
Intrapartum complications		
Fetal heart rate abnormalities	4	72
Meconium-stained liquor	5	58
Oxytocin induction	4	50
Ventouse delivery	4	27
Breech delivery	4	9
Prolonged second stage of labour	3	13
Emergency caesarean section	7	NR
Premature rupture of membranes	6	35
Vaginal blood loss	1	2
Cord abnormality	4	NR
Forceps delivery	2	3
abruption placenta	1	5
Infant characteristics		
Arterial umbilical cord ph	3	26
Apgar score below 7 at 5 min	4	37
Congenital heart disease	1	3
Resuscitation at birth	4	NR

NR: not reported.

### Preeclampsia

**Figure 2:**
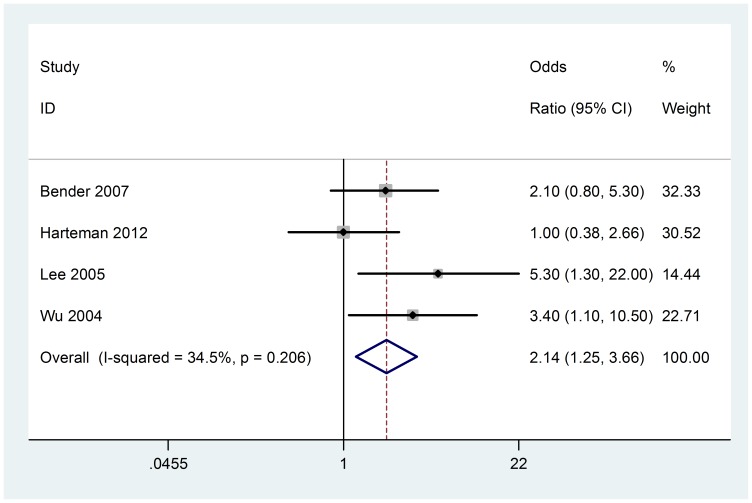
Fixed-effect model forest plot of odds ratio of the association between preeclampsia and AIS. The pooled OR of preeclampsia is symbolized by a solid diamond at the bottom of the forest plot and the width of which represents the 95% CI.

**Figure 3:**
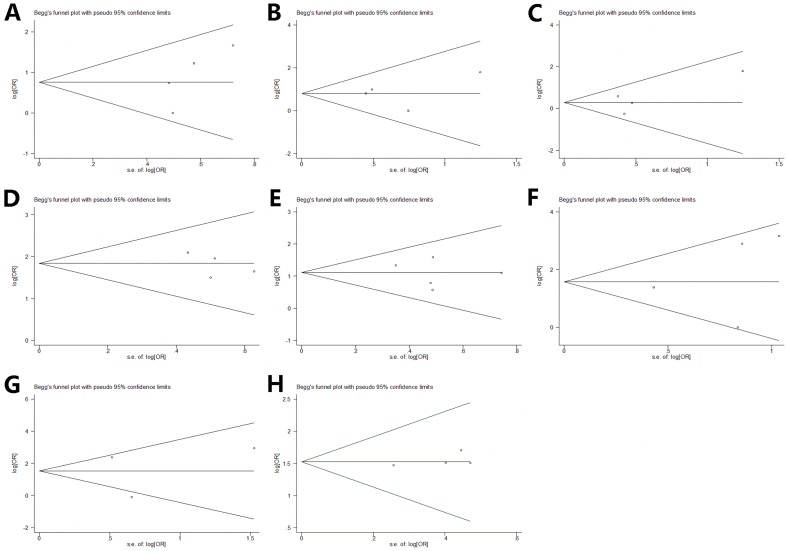
Begg's Funnel plot for publication bias test. The two oblique lines indicate the pseudo 95% confidence limits. a. funnel plot with pseudo 95% CI of preeclampsia b. funnel plot with pseudo 95% CI of ventouse delivery c. funnel plot with pseudo 95% CI of oxytocin induction d. funnel plot with pseudo 95% CI of heart rate abnormalities e. funnel plot with pseudo 95% CI of meconium-stained liquor f. funnel plot with pseudo 95% CI of Apgar scores g. funnel plot with pseudo 95% CI of arterial umbilical cord ph h. funnel plot with pseudo 95% CI of resuscitation at birth.

Four studies (158 cases with AIS) were included in the meta-analysis. No significant heterogeneity was found (Chi^2^ = 3.90, P = 0.272, I^2^ = 23.2%) (Figure 2), so fixed-effect model was used. The association between preeclampsia and AIS were seen in all meta-analyses (OR 2.14; 95% CI, 1.25 to 3.66). Visual assessment of a funnel plot and Beggar's test provided no evidence of obviously publication bias (P = 1.000) (Figure 3).

### Ventouse delivery

**Figure 4:**
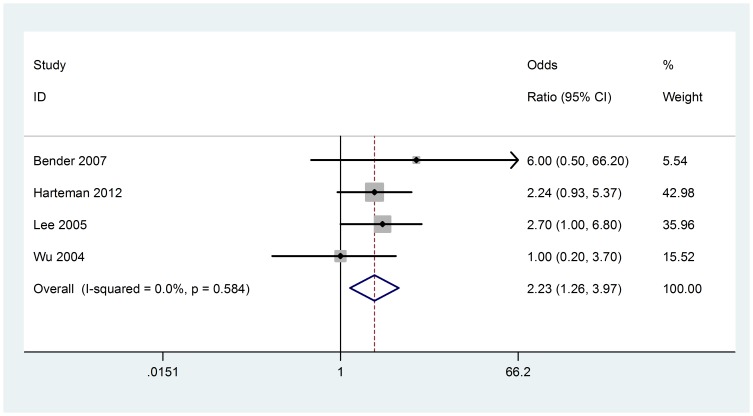
Fixed-effect model forest plot of odds ratio of the association between ventouse delivery and AIS. The pooled OR of ventouse delivery is symbolized by a solid diamond at the bottom of the forest plot and the width of which represents the 95% CI.

Four studies (158 cases with AIS) were included in the meta-analysis. No heterogeneity was found (Chi^2^ = 1.94, P = 0.584, I^2^ = 0%) (Figure 4), we used fixed-effect model. The ventouse delivery is associated with AIS based on the data from the meta-analyses, (OR 2.23; 95% CI, 1.26 to 3.97). Visual assessment of a funnel plot and Beggar's test provided no evidence of obviously publication bias (P = 1.000) (Figure 3).

### Oxytocin induction

**Figure 5:**
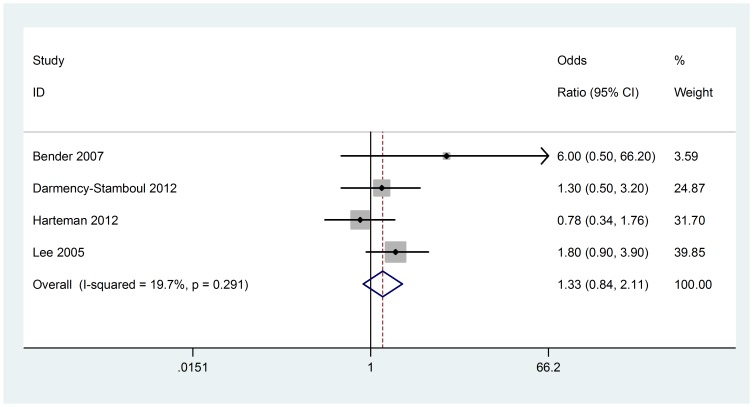
Fixed-effect model forest plot of odds ratio of the association between oxytocin induction and AIS. The pooled OR of oxytocin induction is symbolized by a solid diamond at the bottom of the forest plot and the width of which represents the 95% CI.

Four studies (152 cases with AIS) were included in the meta-analysis. No significant heterogeneity was found (Chi^2^ = 3.74, P = 0.291, I^2^ = 19.7%) (Figure 5), so fixed-effect model was used. Analysis of the data showed that oxytocin induction is associated with AIS (OR 1.33; 95% CI, 0.84 to 2.11). Visual assessment of a funnel plot and Beggar's test provided no evidence of obviously publication bias (P = 1.000) (Figure 3).

### Fetal heart rate abnormalities

**Figure 6:**
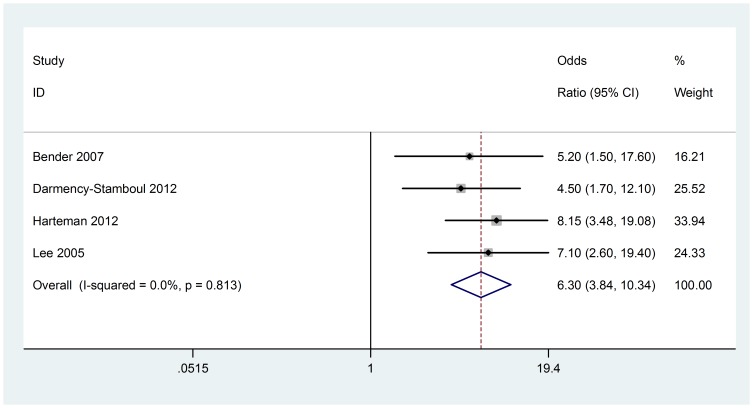
Fixed-effect model forest plot of odds ratio of the association between fetal heart rate abnormalities and AIS. The pooled OR of fetal heart rate abnormalities is symbolized by a solid diamond at the bottom of the forest plot and the width of which represents the 95% CI.

Four case-control studies (152 cases with AIS) were included in the meta-analysis. No heterogeneity was found (Chi^2^ = 0.95, P = 0.813, I^2^ = 0%) (Figure 6), so fixed-effect model was used. The association between fetal heart rate abnormalities and AIS were seen in all meta-analyses (OR 6.3; 95% CI, 3.84 to 10.34). Visual assessment of a funnel plot and Beggar's test provided no evidence of obviously publication bias (P = 1.000) (Figure 3).

### Reduced fetal movement

**Figure 7:**
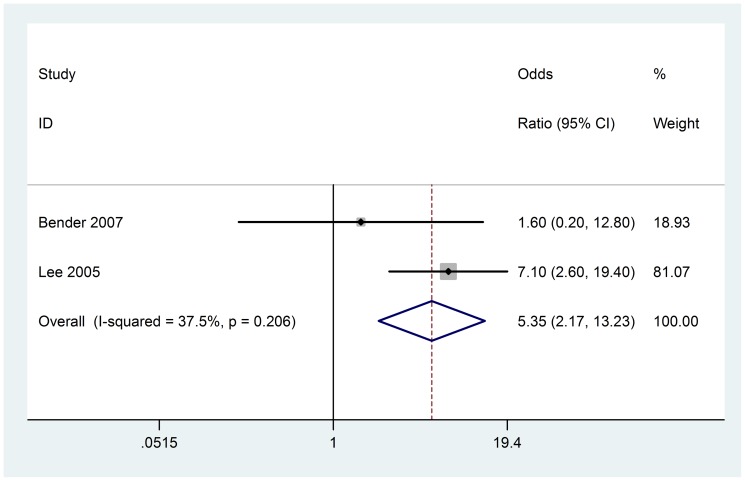
Fixed-effect model forest plot of odds ratio of the association between reduced fetal movement and AIS. The pooled OR of reduced fetal movement is symbolized by a solid diamond at the bottom of the forest plot and the width of which represents the 95% CI.

Two studies (68 cases) included in the meta-analysis. There was not heterogeneity among included studies (Chi^2^ = 0.03, P = 0.867, I^2^ = 0%) (Figure 7), fixed-effect model was used. Analysis of the findings showed that reduced fetal movement is associated with AIS (OR 5.35; 95% CI, 2.17 to 13.23). We did not carry out the test of publication bias because of only two studies.

### Meconium-stained liquor

**Figure 8:**
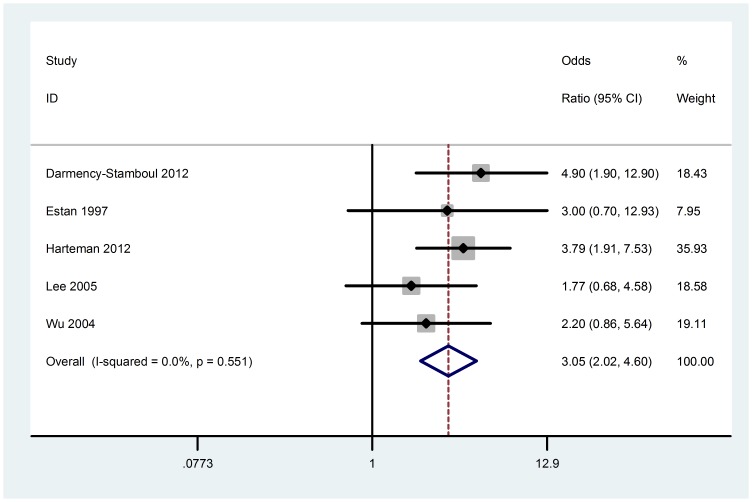
Fixed-effect model forest plot of odds ratio of the association between meconium-stained liquor and AIS. The pooled OR of meconium-stained liquor is symbolized by a solid diamond at the bottom of the forest plot and the width of which represents the 95% CI.

Estimates were reported from five retrospective case–control studies with 171 cases. No heterogeneity was found (Chi^2^ = 3.04, P = 0.551, I^2^ = 0%), we chose fixed-effect model to do the meta-analysis. This meta-analysis showed that meconium-stained liquor is significantly associated with AIS (OR 3.05; 95% CI, 2.02 to 4.60) (Figure 8). Visual assessment of a funnel plot and Beggar's test provided no evidence of obviously publication bias (P = 1.000) (Figure 3).

### Low Apgar score

**Figure 9:**
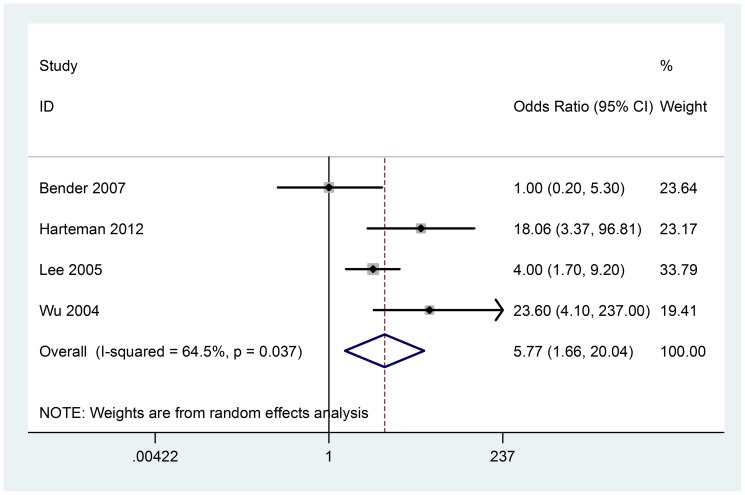
Random-effect model forest plot of odds ratio of the association between low Apgar scores and AIS. The pooled OR of low Appgar scores is symbolized by a solid diamond at the bottom of the forest plot and the width of which represents the 95% CI.

Six studies compared Apgar score between groups, however, Apgar score below 7 at 5 minutes is usually considered asphyxia, and we regarded this as the inclusion standard. Four case-control studies (158 cases) contributed to the meta-analysis. There was significant heterogeneity among included studies (Chi^2^ = 8.46, P = 0.037, I^2^ = 64.5%) (Figure 9), random-effect model was used. Analysis of the findings showed that low Apgar score is associated with AIS (OR 5.77; 95% CI, 1.66 to 20.04). No evidence of obviously publication bias was found according to funnel plot and Beggar's test (P = 1.000) (Figure 3).

### Arterial umbilical cord ph

**Figure 10:**
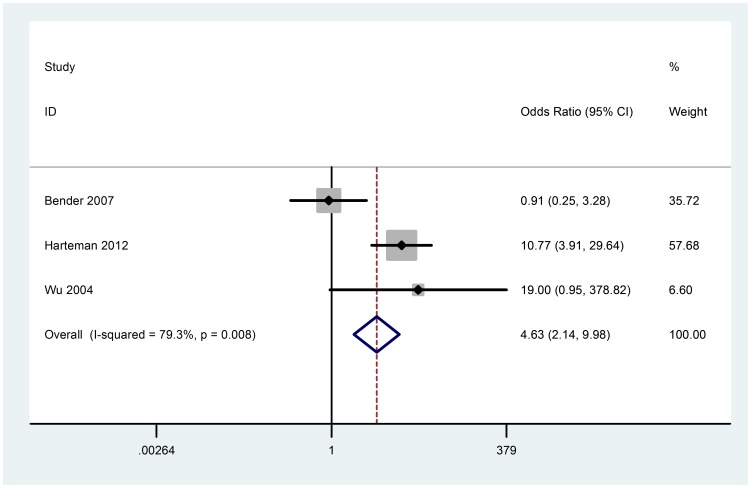
Random-effect model forest plot of odds ratio of the association between arterial umbilical cord ph and AIS. The pooled OR of arterial umbilical cord ph is symbolized by a solid diamond at the bottom of the forest plot and the width of which represents the 95% CI.

Four studies compared arterial umbilical cord ph between groups. Arterial umbilical cord ph below 7.1 is considered mild or moderate academia, we regarded 7.1 as the inclusion standard. Three studies (121 cases) were included in the meta-analysis. There was heterogeneity among included studies (Chi^2^ = 9.66, P = 0.008, I^2^ = 79.3%) (Figure 10), random-effect model was used. Based on the data from the studies, the meta-analysis did not show any significant change of the mean risk estimate (OR 4.63; 95% CI 2.14 to 9.98). No evidence of obviously publication bias was found according to funnel plot and Beggar's test (P = 1.000) (Figure 3).

### Resuscitation at birth

**Figure 11:**
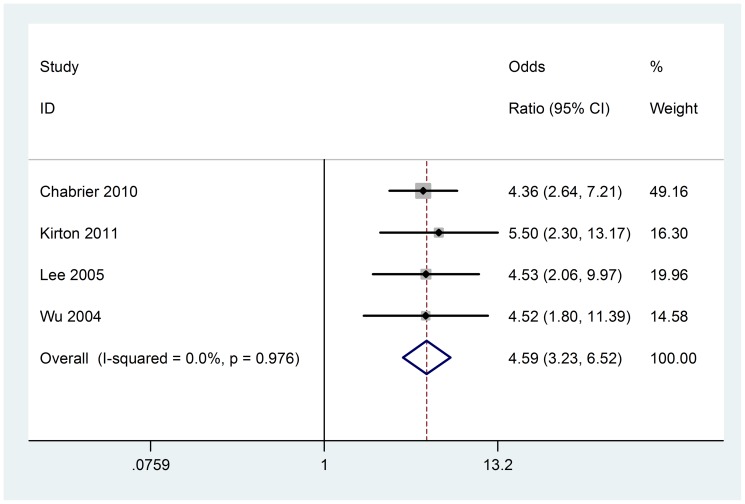
Fixed-effect model forest plot of odds ratio of the association between resuscitation at birth and AIS. The pooled OR of resuscitation at birth is symbolized by a solid diamond at the bottom of the forest plot and the width of which represents the 95% CI.

Three retrospective and one prospective case-control studies (423 cases) contributed to the meta-analysis. There was not heterogeneity among included studies (Chi^2^ = 0.21, P = 0.976, I^2^ = 0%) (Figure 11), fixed-effect model was used. Analysis of the findings showed that resuscitation at birth is associated with AIS (OR 4.59; 95% CI, 3.23 to 6.52). No evidence of obviously publication bias was found according to funnel plot and Beggar's test (P = 1.000) (Figure 3).

## Discussion

Our Study included 8 studies and 550 newborns with AIS. The pooled analysis indicates that the associations were found for AIS: preeclampsia (OR 2.14; 95% CI, 1.25 to 3.66), ventouse delivery (OR 2.23; 95% CI, 1.26 to 3.97), fetal heart rate abnormalities (OR 6.3; 95% CI, 3.84 to 10.34), reduced fetal movement (OR 5.35; 95% CI, 2.17 to 13.23), meconium-stained liquor (OR 3.05; 95% CI, 2.02 to 4.60), low Apgar score (OR 5.77; 95% CI, 1.66 to 20.04) and resuscitation at birth (OR 4.59; 95% CI, 3.23 to 6.52). Our data did not show any significant change of the mean risk estimate for oxytocin induction (OR 1.33; 95% CI, 0.84 to 2.11) and low arterial umbilical cord ph (OR 4.63; 95% CI 2.14 to 9.98).

Neonatal arterial ischemic stroke is the most common form of cerebral infarction in children, and a leading cause of lifelong neurodevelopmental disabilities. Although the diagnosis is easily confirmed by neuro-imaging [24], about half the children arouse no concern during the neonatal period. However, when neonates are unstable, neuro-imaging can be difficult, particularly MRI and CT. In neonates, cranial ultrasound is a noninvasive and easily accessible intervention for the evaluation of stroke [25]. But it may not accurately diagnose stroke, (the cranial ultrasound is known to miss smaller cortical infarcts), especially since these events do not necessarily lead to neurologic symptoms later in infancy [20]. So identifying newborns at high risk of AIS is important.

The preeclampsia usually is associated with abnormalities in the development of placental vessels. And hypoxia is one of the critical components in the pathogenesis of preeclampsia [26]. Birth asphyxia was more common in the ventouse delivery cohort compared with normal delivery [27]. Our study suggests that preeclampsia and ventouse delivery may be one of the risk factors of AIS. Appropriate use of oxytocin induction generally may not lead to neonatal hypoxia. Our study did not show any association between oxytocin induction and AIS.

Fetal heart rate abnormalities and reduced fetal movement have been routinely considered as predictors of fetal distress. As meconium passage is associated with reduced cerebral artery pulsatility index, some evidence suggests that meconium-stained amniotic fluid may reflect fetal hypoxia [28]. Analysis of our results shows that for studies evaluating AIS, fetal heart rate abnormalities, reduced fetal movement and meconium-stained amniotic fluid may be risk factors.

Apgar score is a sum of values assigned to an infant, with a score of 7 or more indicating that the baby is in good to excellent condition [29]. An Apgar score <7, suggestive of fetal distress, is more commonly associated with AIS than previously reported, according to our meta-analysis. But because of the heterogeneity, further study is needed to confirm this conclusion. We speculate that the reason may be the clinical heterogeneity, one study took the Apgar score <5 at 5 min as the inclusion criteria [20], while the rest studies took the Apgar score <7 at 5 min as the inclusion criteria. Low Apgar score likely reflects adverse events during delivery and suggests an important role for subsequent hypoxia-ischemia in the pathogenic pathway of AIS [14].

In most centers, an umbilical cord ph of 7 to 7.1 is considered mild or moderate academia, with severe acidemia occurring when the ph is below 7. It has been proposed that neonatal complications are associated with metabolic rather than respiratory acidosis [30]. Respiratory acidosis arises in the early stages of impaired blood supply to the fetus, with a subsequent reduction in the cord ph [31]. Other studies suggest that neurologic injury is more likely to occur in an infant who is distressed but has a normal ph [32]. Hermansen suggested there is an acidosis paradox, or a beneficial effect of a mild to moderate acidosis [33]. We did not find any association between umbilical cord ph and AIS. But as measurement of umbilical cord ph is not part of routine care in some obstetrics facilities, we only had these data in three studies. There was heterogeneity among these studies, the reason may be the clinical and statistical heterogeneity, one study included preterm newborns [20], full term was admitted to another [14]; one used univariable analysis [14], another used multivariable analysis [20]. Thus, more clinical studies are required to assess the relationship between arterial umbilical cord ph and AIS.

Resuscitation at birth usually refers to intubation, assisted ventilation, chest compressions, or cardiac medication. Neonates had transient hypoxia that needed resuscitation at birth. Kirton found that children with arterial presumed perinatal ischemic stroke were more likely to have neonatal resuscitation [34], which was consistent with ours.

Preeclampsia and ventouse delivery are intrapartum complications, both may cause neonatal hypoxia. Fetal heart rate abnormalities reduced fetal movement, meconium-stained amniotic fluid, low Apgar score and resuscitation at birth, all of which are recognized markers of perinatal hypoxia. All of them were more common in AIS cases than in control subjects. Our results suggested a role of perinatal hypoxia in the development of AIS. The same characteristics of neonatal distress suggest that shared pathological mechanisms for perinatal stroke and neonatal encephalopathy may exist, and both pathologies can furthermore coexist in the same baby, as shown by Ramaswamy [17].

AIS and global hypoxic-ischemic encephalopathy likely share risk factors and AIS can occur alongside hypoxic-ischemic encephalopathy. In Wu et al, 17% of infants with AIS had a low Apgar score, but 0.9% from a normal cohort had a low Apgar score [9].

Our findings imply that AIS is more often associated with a complicated gestational course and delivery, resulting in fetal distress or hypoxia. Since the occurrence of stroke in this cohort was more frequent than in population-based data, neuro-imaging should be performed in these infants. And the study comparing neonates with seizures caused by stroke with seizures caused by HIE reported that seizure onset >12 h and clinically observed focal seizures are more often associated with AIS [35]. So our findings suggest the use of MRI as the first-line imaging modality in most cases of neonatal encephalopathy, which facilitates the accurate diagnosis of AIS [23].

Although there is data about the risk factors for AIS, knowledge is still limited. In our meta-analysis, most of the included studies were designed retrospectively. Given the retrospective nature of these studies, it follows that bias towards risk factors may have occurred in the more severe cases [22]. The relatively small number of cases in subgroups resulted in statistical imprecision, and the results should accordingly be interpreted with caution. This study was subject to a number of limitations. However, there is a significant association between perinatal hypoxia factors, likewise preeclampsia, ventouse delivery, fetal heart rate abnormalities, reduced fetal movement, meconium-stained liquor, low Apgar score, resuscitation at birth and AIS. The results indicate that perinatal hypoxia maybe one of causes of AIS. Large scale prospective clinical studies are still warranted.

## Supporting Information

File S1 **PRISMA Checklist.**
(DOC)
