# Supplementary material for: Association between Hypoxia and Perinatal Arterial Ischemic Stroke: A Meta-Analysis
Source: PLoS One. 2014 Feb 28;9(2):e90106. doi: 10.1371/journal.pone.0090106 (PMC3938587; doi:10.1371/journal.pone.0090106)
Supplement: File S1 — PRISMA Checklist. (DOC) [file pone.0090106.s001.doc]

| **Section/topic** | **#** | **Checklist item** | **Reported on page #** |
| --- | --- | --- | --- |
| **TITLE** | | |  |
| Title | 1 | Association between Hypoxia and Perinatal Arterial Ischemic Stroke: A meta-analysis | Title |
| **ABSTRACT** | | |  |
| Structured summary | 2 | Background: Perinatal arterial ischemic stroke (AIS) occurs in an estimated 17 to 93 per 100000 live births, yet the etiology is poorly understood. Although investigators have implicated hypoxia as a potential cause of AIS, the role of hypoxia in AIS remains controversial. The aim of this study was to estimate the association between perinatal hypoxia factors and perinatal arterial ischemic stroke through a meta-analysis of published observational studies. Patients and methods: A systematic search of electronically available studies published through July 2013 was conducted. Publication bias and heterogeneity across studies were evaluated and summary odds ratios (ORs) and 95% confidence intervals (CIs) were calculated with fixed-effects or random-effects models. Results: A total of 8 studies describing the association between perinatal hypoxia factors and neonatal arterial ischemic stroke (AIS) met inclusion criteria, and 550 newborns with AIS were enrolled. The associations were found for AIS: preeclampsia (OR 2.14; 95% CI, 1.25 to 3.66), ventouse delivery (OR 2.23; 95% CI, 1.26 to 3.97), fetal heart rate abnormalities (OR 6.30; 95% CI, 3.84 to 10.34), reduced fetal movement (OR 5.35; 95% CI, 2.17 to 13.23), meconium-stained liquor (OR 3.05; 95% CI, 2.02 to 4.60), low Apgar score (OR 5.77; 95% CI, 1.66 to 20.04) and resuscitation at birth (OR 4.59; 95% CI, 3.23 to 6.52). Our data did not show any significant change of the mean risk estimate for oxytocin induction (OR 1.33; 95% CI, 0.84 to 2.11) and low arterial umbilical cord ph (OR 4.63; 95% CI 2.14 to 9.98).  CONCLUSIONS: There is a significant association between perinatal hypoxia factors and AIS. The result indicates that perinatal hypoxia maybe one of causes of AIS. Large scale prospective clinical studies are still warranted. | Abstract |
| **INTRODUCTION** | | |  |
| Rationale | 3 | Perinatal ischemic stroke is defined as a group of heterogeneous conditions in which there is a focal disruption of cerebral blood flow secondary to arterial or venous thrombosis or embolization, which occurred from birth up until 28 days postnatal.  The prevalence of PAIS has not been clearly determined. Previous estimates range from 17 to 93 per 100 000 live births. Perinatal arterial ischemic stroke is a main cause of cerebral palsy and other neurologic disabilities, thus making it a clinically relevant type of brain injury, yet the etiology is poorly understood.  The pathogenesis of AIS is complex and multifactorial. Risk factors may be related to both maternal and placental problems as well as fetal and neonatal disorders. Investigators have implicated hypoxia as a potential cause of AIS. In a series of 250 newborns examined in the 1980s and 1990s, 35% of strokes occurred in the context of perinatal asphyxia. Some term newborns were found with hypoxic-ischemic encephalopathy, particularly in cases of arterial infarction, (stroke). In a prospective cohort study of 124 term newborns with hypoxia-ischemia encephalopathy, 6 neonates had arterial stroke identiﬁed by neuroimaging. Although there was an increased incidence of hypoxia factors in the stroke group, some studies reported there was no signiﬁcant difference between groups, the information published to date is conflicting. | Introduction |
| Objectives | 4 | The objective of this meta-analysis was to determine the impact of clinical risk factors or markers for hypoxia in neonates with arterial stroke. Our hypothesis is that clinical risk factors for hypoxia differ between newborns with stroke compared to normal controls | Introduction |
| **METHODS** | | |  |
| Protocol and registration | 5 | This research use the PRISMA (Preferred Reporting Items for Systematic Reviews and Meta-Analyses) statement as a guide.(available at：http://www.prisma-statement.org) | Methods and Patients |
| Eligibility criteria | 6 | Case-control and cohort studies. | Methods and Patients |
| Information sources | 7 | Studies were searched in databases PubMed, EMBASE, Web of Science and Cochrane Library from inception to July 2013. | Methods and Patients |
| Search | 8 | The search terms were perinatal stroke, perinatal and neonatal ischemic stroke, neonatal arterial ischemic stroke, fetal stroke, and presumed prenatal or perinatal arterial ischemic stroke; prepartum complications, intrapartum complications and risk factor. Search consisted of a combination of one term from each group. The manual search was applied in the reference of included studies. | Methods and Patients |
| Study selection | 9 | Studies that met the following criteria were included: (1) Studies included should be published, regardless of its design, publication language or date. (2)The studies comparing risk factors between neonates with AIS and control subjects. (3) stroke cases were objectively confirmed by suitable imaging methods (CT and/or MR scan)  Case reports and case series/studies lacking these controls were excluded, as were cases of cerebral sinovenous thrombosis or hemorrhagic stroke. | Methods and Patients |
| Data collection process | 10 | Data extraction was conducted independently by 2 investigators and Quality of the studies was assessed using the Newcastle-Ottawa Quality Assessment Scale for case-control and cohort studies by 2 independent evaluators. Data were adjudicated by 2 additional investigators according to the original articles after data extraction and assessment. Any disagreement will be present to discuss within all authors. | Methods and Patients |
| Data items | 11 | Characteristics of studies (author name, publication time, study design, sample size, birth weight) and outcomes such as odds ratios were collected. If original odds ratio was not reported it will be calculated according to the original data. | Methods and Patients |
| Risk of bias in individual studies | 12 | The report quality of studies was assessed using the Newcastle-Ottawa Quality Assessment Scale for case-control and cohort studies, including patient selection, study comparability and outcome by 2 independent evaluators. This scale is an eight-item instrument that allows for assessment of patient population and selection, study comparability, follow-up, and outcome of interest. Interpretation of the scale is performed by awarding points, or stars’, for high-quality elements. Stars are then added up and used to compare study quality in a quantitative manner. | Methods and Patients |
| Summary measures | 13 | Odds Ratio, 95% confidence intervals (CIs). | Methods and Patients |
| Synthesis of results | 14 | The association between each risk factor and AIS was expressed as odds ratio (OR).  All statistical analysis was performed using Stata 12.0. The pooled OR and its 95% confidence intervals (CIs) were calculated using either fixed-effect model or random-effect model. A fixed-effect model was used when heterogeneity was not detected (P>0.10); otherwise, a random-effect model was used. For quantitative evaluation, OR was used to estimate the impact of risk factors on incidence of AIS. OR, variance, 95% CI, log(OR) and se(log(OR)) for each study were extracted or calculated by Stata 12.0 based on the published studies. The weight for each study is the inverse of study variance and variability between studies. A significant two-way P value for comparison was defined as P<0.05. Results are described using forest plots, where a single square represents an individual study’s OR estimate. The pooled OR is symbolized by a solid diamond at the bottom of the forest plot and the width of the square represents the 95% CI of the OR. The size of the square represents the weight that the corresponding study exerts in the meta-analysis.  Statistical heterogeneity between studies was examined using both the Cochrane Q statistic (significant at P<0.1) and the I^2^ value. I^2^>50% were considered to represent significant heterogeneity. Assessment of publication bias was performed for each of the pooled study groups using the Bgger’s bias indicator test. The analysis of publication bias was carried out using the statistical software Stata version12.0. | Methods and Patients |

Page 1 of 2

| **Section/topic** | | | **#** | | **Checklist item** | **Reported on page #** |
| --- | --- | --- | --- | --- | --- | --- |
| Risk of bias across studies | | | 15 | | The potential publication bias was evaluated by funnel plots and Beggar’s bias indicator test. | Methods and Patients |
| Additional analyses | | | 16 | | No subgroup and additional analysis exists. | Methods and Patients |
| **RESULTS** | | | | | |  |
| Study selection | | 17 | | Fig 1. Flow chart of the search result of the meta-analysis. | | Result |
| Study characteristics | | 18 | | | **Study** | **Years** | **Country** | **Study type** | **Study design** | **Case number** | **Controls number** | **Neonate** | **Birth weight** | **Imaging confirm** | Risk of bias | | --- | --- | --- | --- | --- | --- | --- | --- | --- | --- | --- | | Benders 2007 [20] | 1990-2005 | US | retrospective | case-control | 31 | 93 | preterm | Cases:1599**±**633  Controls:1607**±**720 | MRI | different detecting method, only preterm neonates | | Chabrier 2010 [21] | 2003-2006 | France | prospective | cohort study | 100 | 100 | both | NR | CT/MRI | different detecting method, no description of those lost to follow up | | Darmency-  Stamboul 2012 [22] | 2000-2007 | France | retrospective | case-control | 32 | 96 | term | NR | CT /MRI | different detecting method, only term neonates | | Estan 1997 [8] | 1987-1993 | UK | retrospective | case-control | 12 | 24 | term | Cases:3496(2541-4460)  Controls:3673 (2748-4707) | CT | different detecting method, only term neonates | | Harteman 2012 [14] | 2000-2010 | Utrecht | retrospective | case-control | 52 | 156 | term | Cases:3420(2145-5230)  Controls:3520 (2155- 4925) | MRI | different detecting method, only term neonates | | Lee 2005 [6] | 1997-2002 | US | retrospective | nested case-control | 37 | 111 | both | Cases:3127**±**852  Controls:3203**±** 923 | CT /MRI | different detecting method | | Wu 2004 [9] | 1991-1998 | US | retrospective | nested case-control | 38 | 218 | term | NR | CT/MRI | different detecting method, only term neonates | | Kirton 2011 [23] | 2003-2007 | IPSS | prospective | case-control | 248 | population | both | NR | CT/MRI | different detecting method |   IPSS: Europe, Canada, US, South America, Asia, Australia  6. Lee J, Croen LA, Backstrand KH, Yoshida CK, Henning LH, et al. (2005) Maternal and infant characteristics associated with perinatal arterial stroke in the infant. JAMA. 293(6):723-729.  8. Estan J, Hope P (1997) Unilateral neonatal cerebral infarction in full term infants. Arch Dis Child Fetal Neonatal Ed. 76(2):F88-93.  9. Wu YW, March WM, Croen LA, Grether JK, Escobar GJ, Newman TB (2004) Perinatal stroke in children with motor impairment: a population-based study. Pediatrics. 114(3):612-619.  14. Harteman JC, Groenendaal F, Kwee A, Welsing PM, Benders MJ, de Vries LS (2012) Risk factors for perinatal arterial ischaemic stroke in full-term infants: a case-control study. Arch Dis Child Fetal Neonatal Ed. 97(6):F411-6.  20. Benders MJ, Groenendaal F, Uiterwaal CS, Nikkels PG, Bruinse HW, Nievelstein RA, de Vries LS (2007) Maternal and infant characteristics associated with perinatal arterial stroke in the preterm infant. Stroke. 38(6):1759-1765.  22. Darmency-Stamboul V, Chantegret C, Ferdynus C, Mejean N, Durand C, Sagot P, Giroud M, Bejot Y, Gouyon JB (2012) Antenatal factors associated with perinatal arterial ischemic stroke. Stroke. 43(9):2307-2312.  21. Chabrier S, Saliba E, Nguyen The Tich S, Charollais A, Varlet MN, et al. (2010) Obstetrical and neonatal characteristics vary with birth weight in a cohort of 100 term newborns with symptomatic arterial ischemic stroke. Eur J Paediatr Neurol. 14(3):206-13.  23. Kirton A, Armstrong-Wells J, Chang T, Deveber G, Rivkin MJ, et al. (2011) Symptomatic neonatal arterial ischemic stroke: the International Pediatric Stroke Study. Pediatrics. 128(6):e1402-1410. | | Result |
| Risk of bias within studies | | 19 | | | Study(year) | Quality score | Risk of bias | | --- | --- | --- | | Benders 2007 [20] | 6 star | different detecting method, only preterm neonates | | Chabrier 2010 [21] | 7 star | different detecting method, no description of those lost to follow up | | Darmency-Stamboul 2012 [22] | 7 star | different detecting method, only term neonates | | Estan 1997 [8] | 6 star | different detecting method, only term neonates | | Harteman 2012 [14] | 6 star | different detecting method, only term neonates | | Lee 2005 [6] | 7 star | different detecting method | | Wu 2004 [9] | 6 star | different detecting method, only term neonates | | Kirton 2011 [23] | 7 star | different detecting method | | Citations see Item 18 | | | | | Result |
| Results of individual studies | | 20 | | 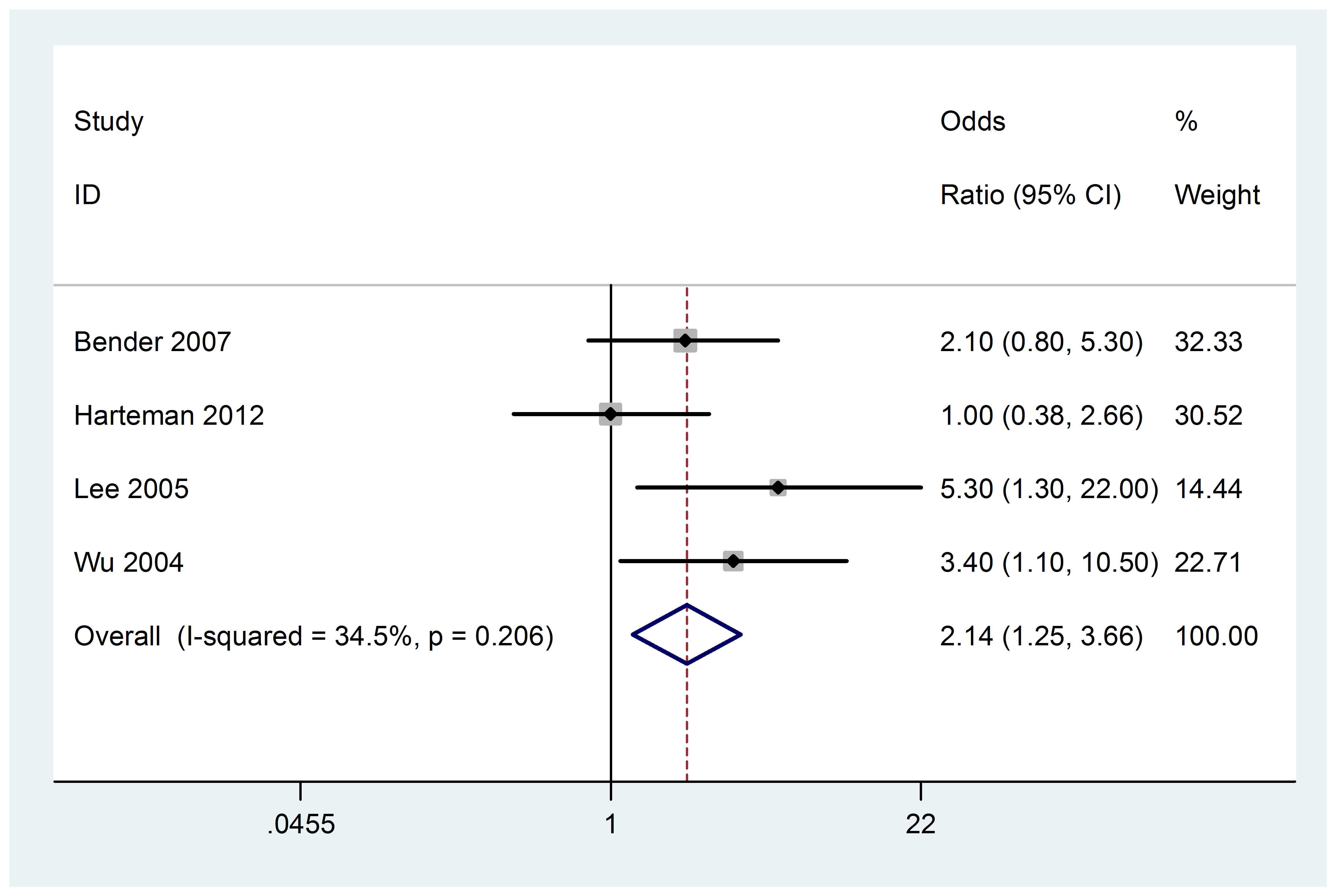  Figure 2. The association between preeclampsia and AIS.  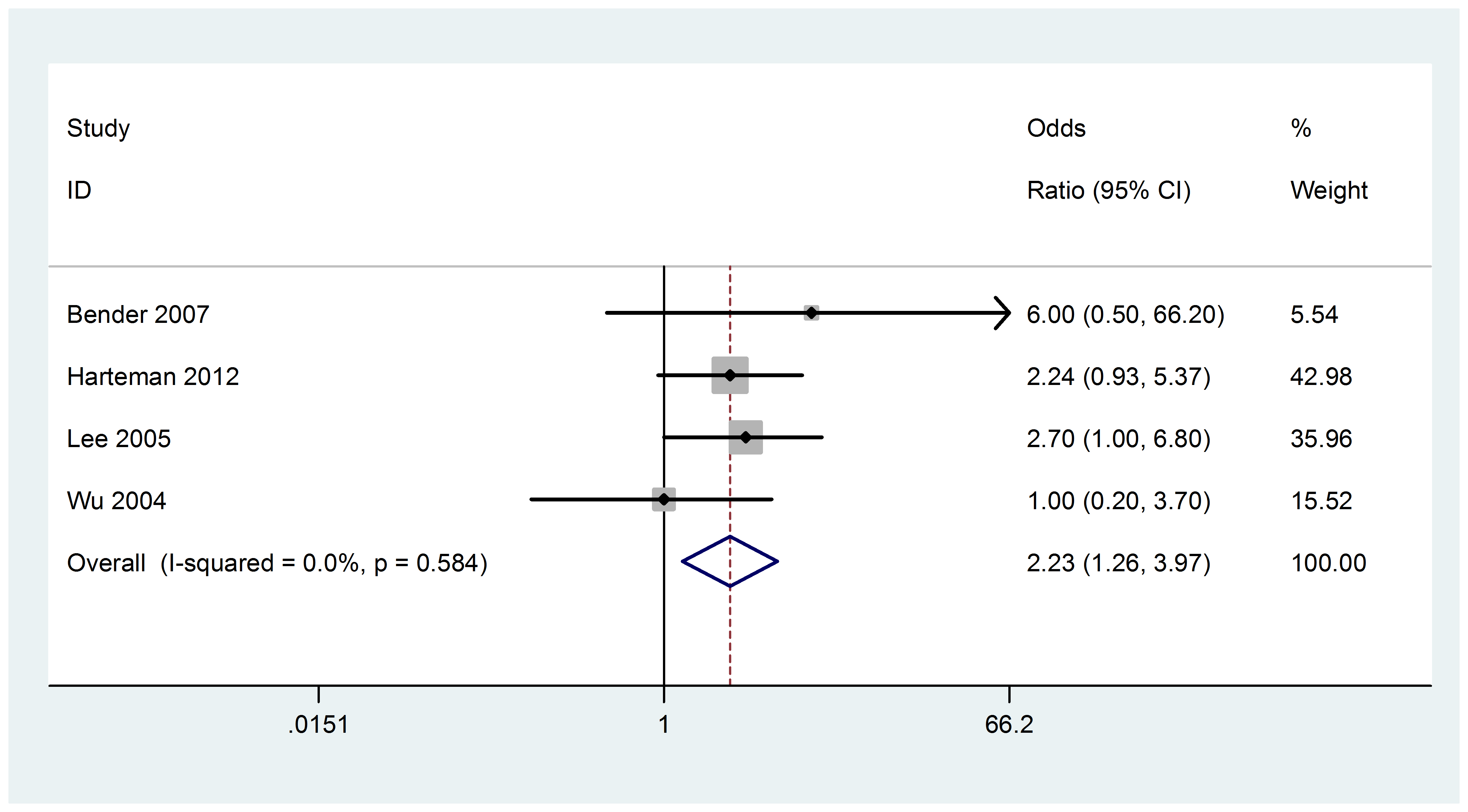  Figure 3. The association between ventouse delivery and AIS.  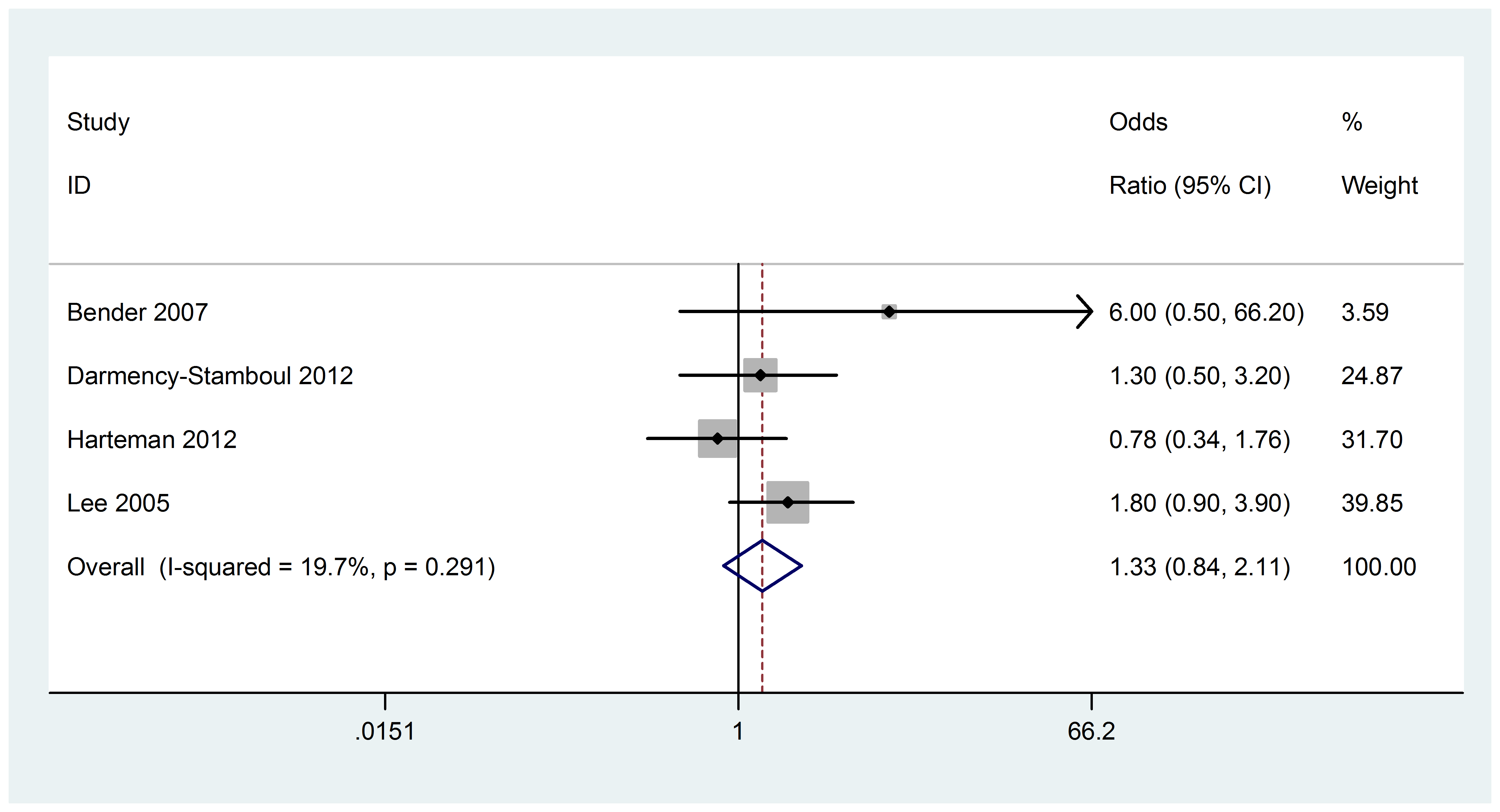  Figure 4. The association between oxytocin induction and AIS.  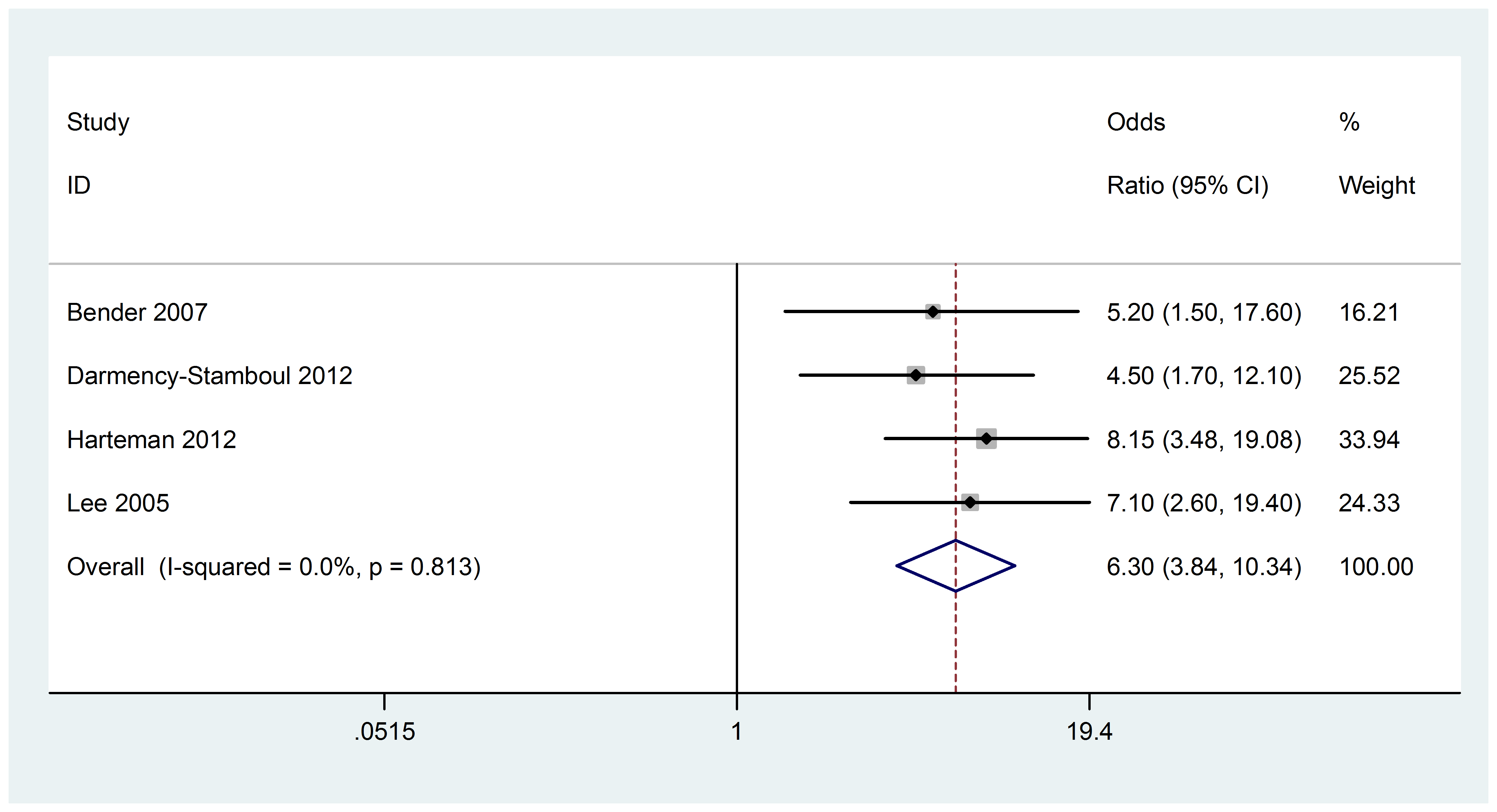  Figure 5. The association between Fetal heart rate abnormality and AIS.  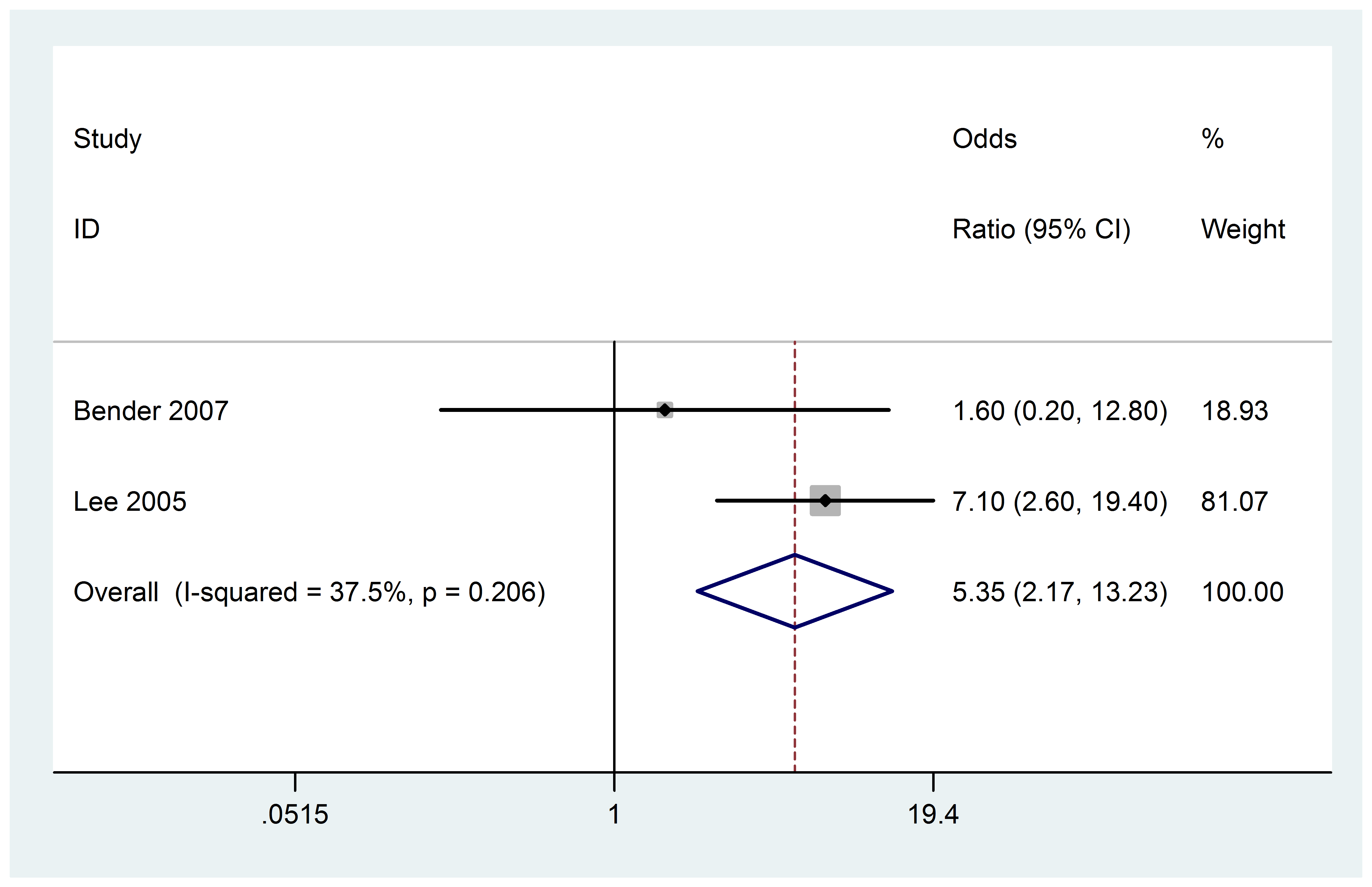  Figure 6. The association between reduced fetal movement and AIS.  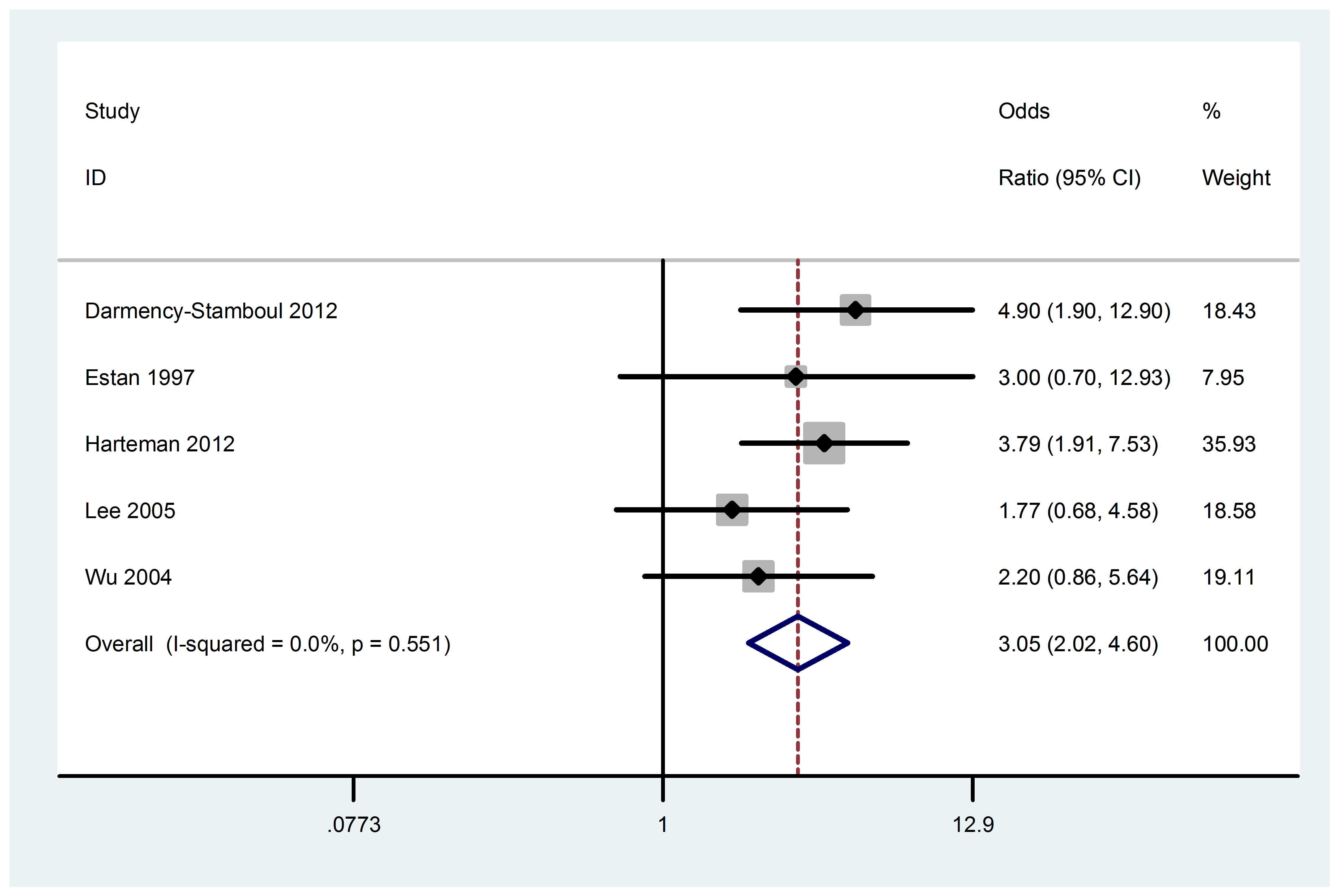  Figure 7. The association between meconium-stained liquor and AIS  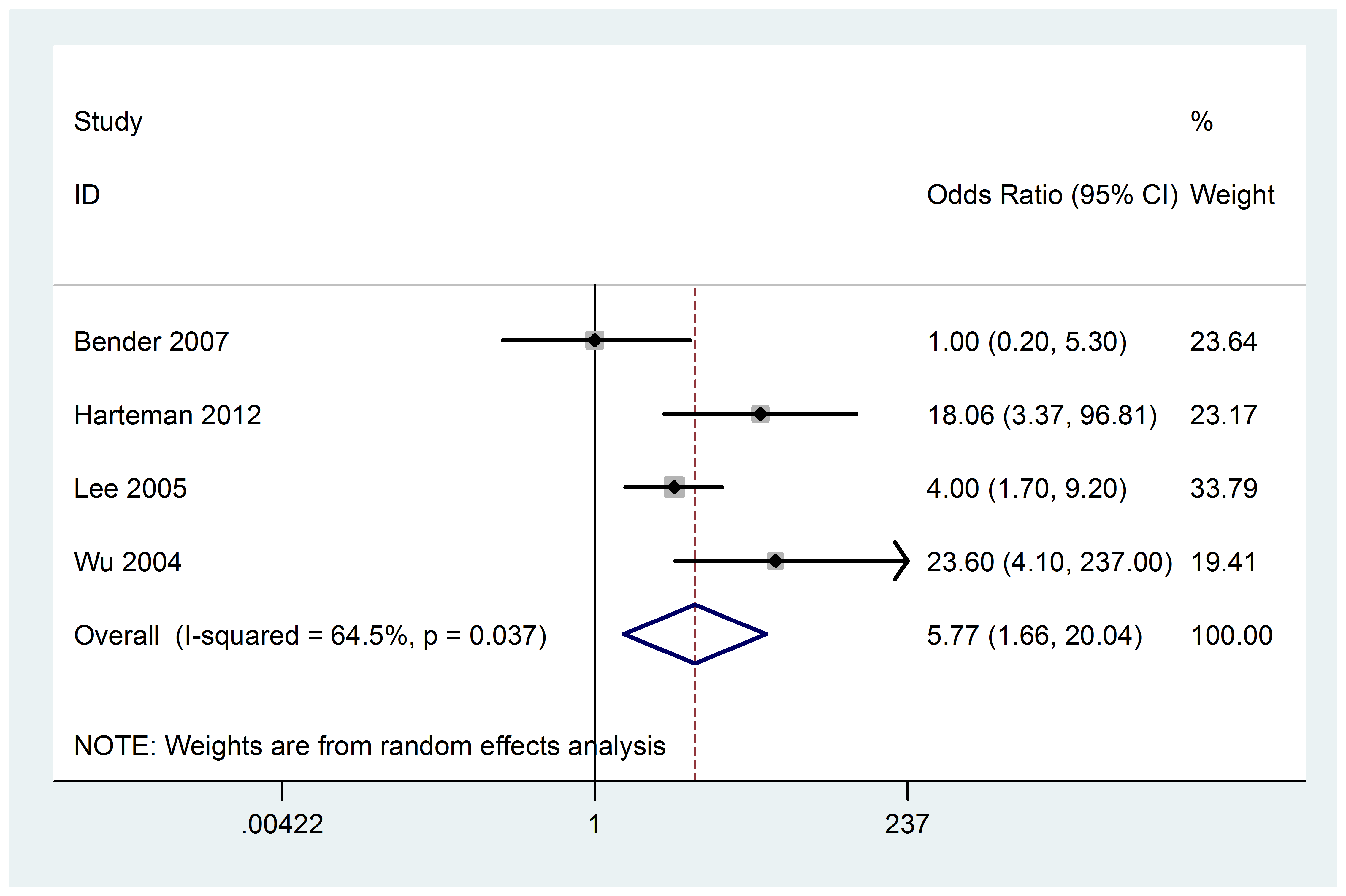  Figure 8. The association between Low Apgar score and AIS  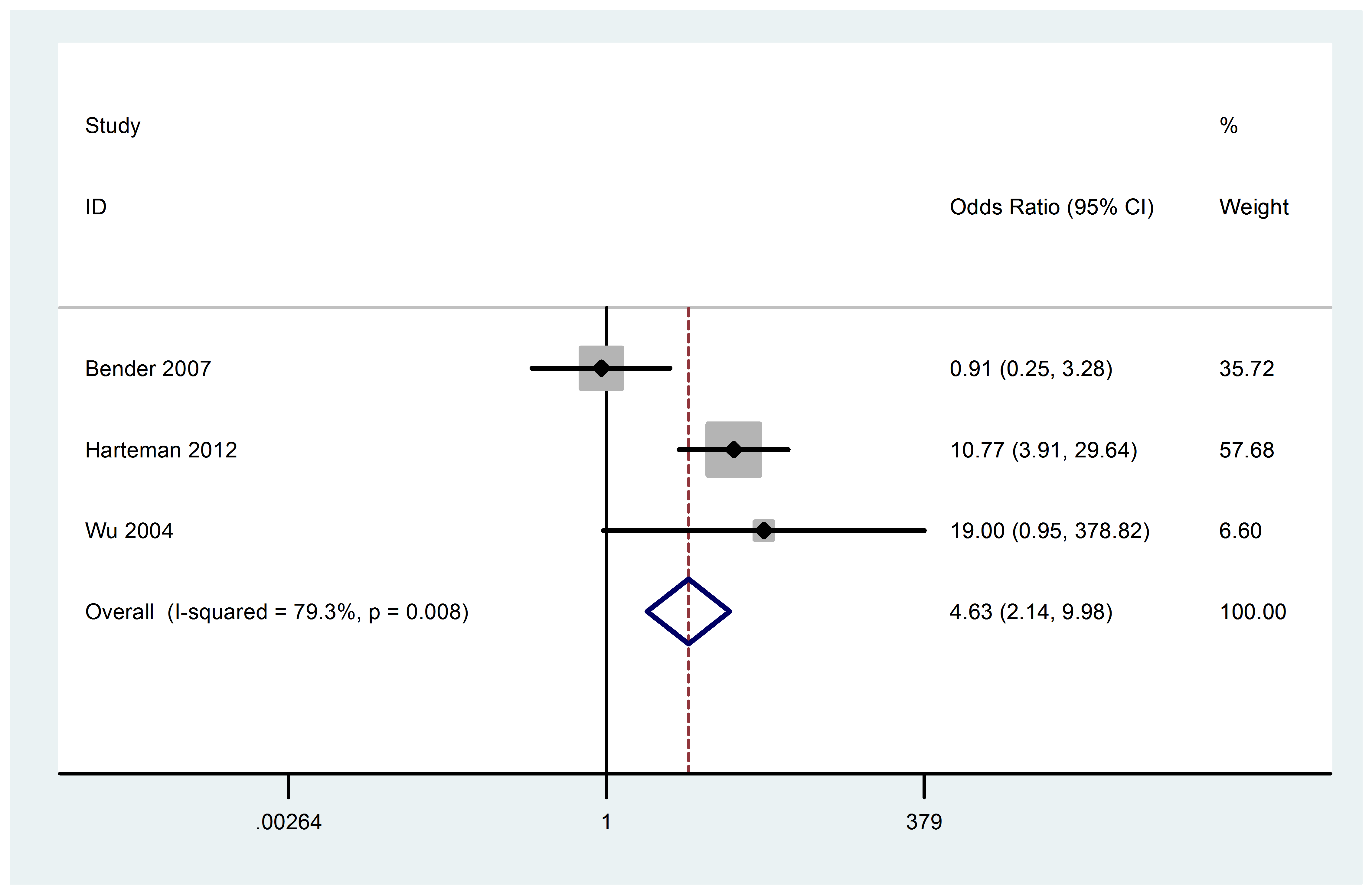  Figure 9. The association between Arterial umbilical cord ph and AIS  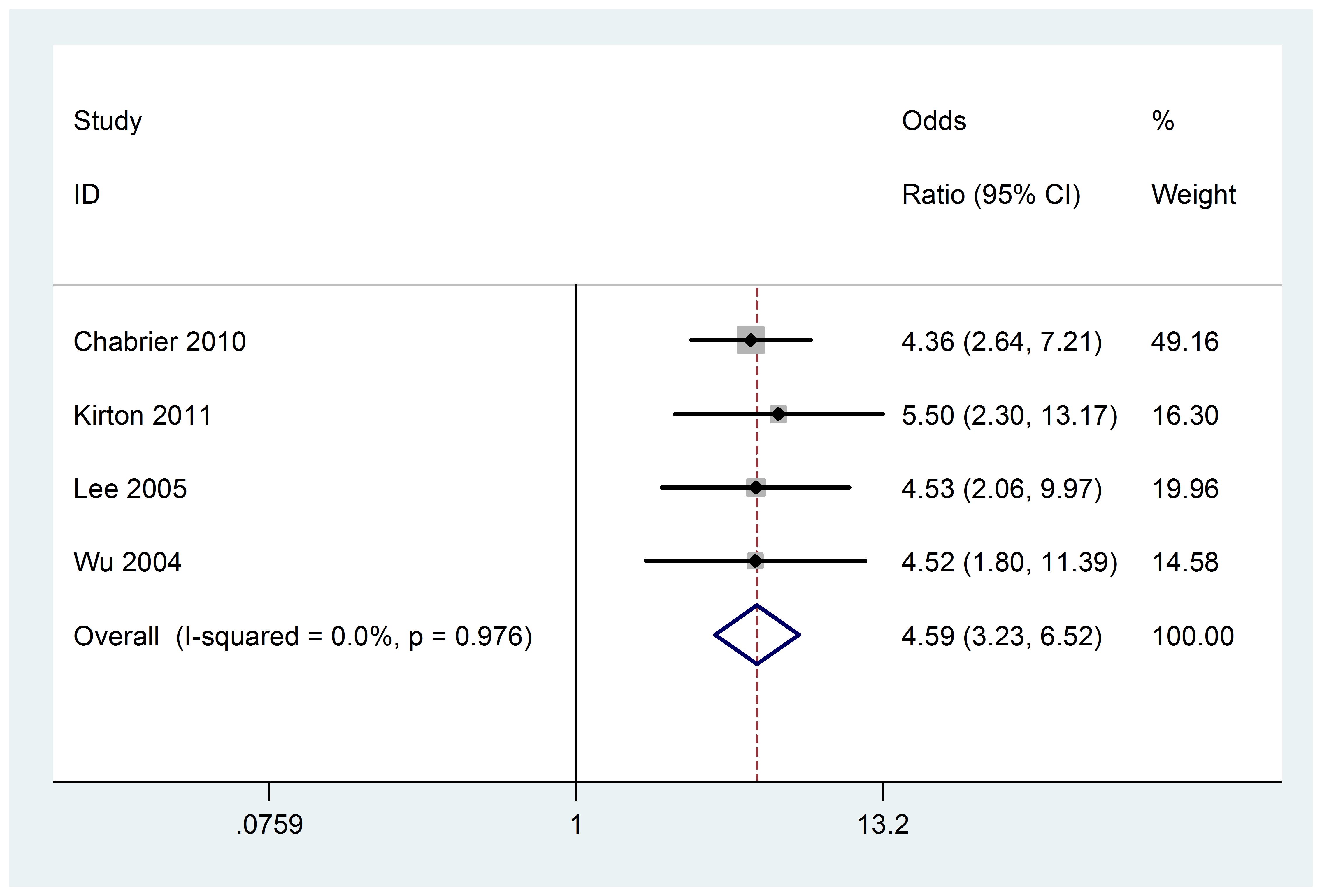  Figure 10. The association between resuscitation at birth and AIS. | | Result |
| Synthesis of results | | 21 | | **Preeclampsia:** The association between preeclampsia and AIS were seen in all meta-analyses (OR 2.14; 95% CI, 1.25 to 3.66). No significant heterogeneity was found (Chi^2^=3.90, P=0.272, I^2^= 23.2%) (Figure 2).  **Ventouse delivery:** The ventouse delivery is associated with AIS based on the data from the meta-analyses, (OR 2.23; 95% CI, 1.26 to 3.97). No heterogeneity was found (Chi^2^=1.94, P=0.584, I^2^= 0%) (Figure 3).  **Oxytocin induction:** oxytocin induction is associated with AIS (OR 1.33; 95% CI, 0.84 to 2.11). No significant heterogeneity was found (Chi^2^=3.74, P=0.291, I^2^= 19.7%) (Figure 4).  **Fetal heart rate abnormalities:**The association between fetal heart rate abnormalities and AIS were seen in the meta-analysis (OR 6.3; 95% CI, 3.84 to 10.34). No heterogeneity was found (Chi^2^=0.95, P=0.813, I^2^= 0%) (Figure 5).  **Reduced fetal movement:** Reduced fetal movement is associated with AIS (OR 6.80; 95% CI, 2.86 to 16.14). There wasn’t heterogeneity among included studies (Chi^2^=0.03, P=0.867, I^2^=0%) (Figure 6).  **Meconium-stained liquor:** Meconium-stained liquor is significantly associated with AIS (OR 3.05; 95% CI, 2.02 to 4.60) (Figure 4). No heterogeneity was found (Chi^2^=3.04, P=0.551, I^2^= 0%) (Figure 7).  **Low Apgar score:** Low Apgar score is associated with AIS (OR 5.77; 95% CI, 1.66 to 20.04). There was significant heterogeneity among included studies (Chi^2^=8.46, P=0.037, I^2^=64.5%) (Figure 8).  **Arterial umbilical cord ph:** The meta-analysis did not show any significant change of the mean risk estimate (OR 4.63; 95% CI 2.14 to 9.98). There was heterogeneity among included studies (Chi^2^=9.66, P=0.008, I^2^=79.3%) (Figure 9).  **Resuscitation at birth:** Resuscitation at birth is associated with AIS (OR 4.59; 95% CI, 3.23 to 6.52). There wasn’t heterogeneity among included studies (Chi^2^=0.21, P=0.976, I^2^=0%) (Figure 10). | | Result |
| Risk of bias across studies | | 22 | | No evidence of obviously publication bias was found according to funnel plot and Beggar’s test (Figure 11).  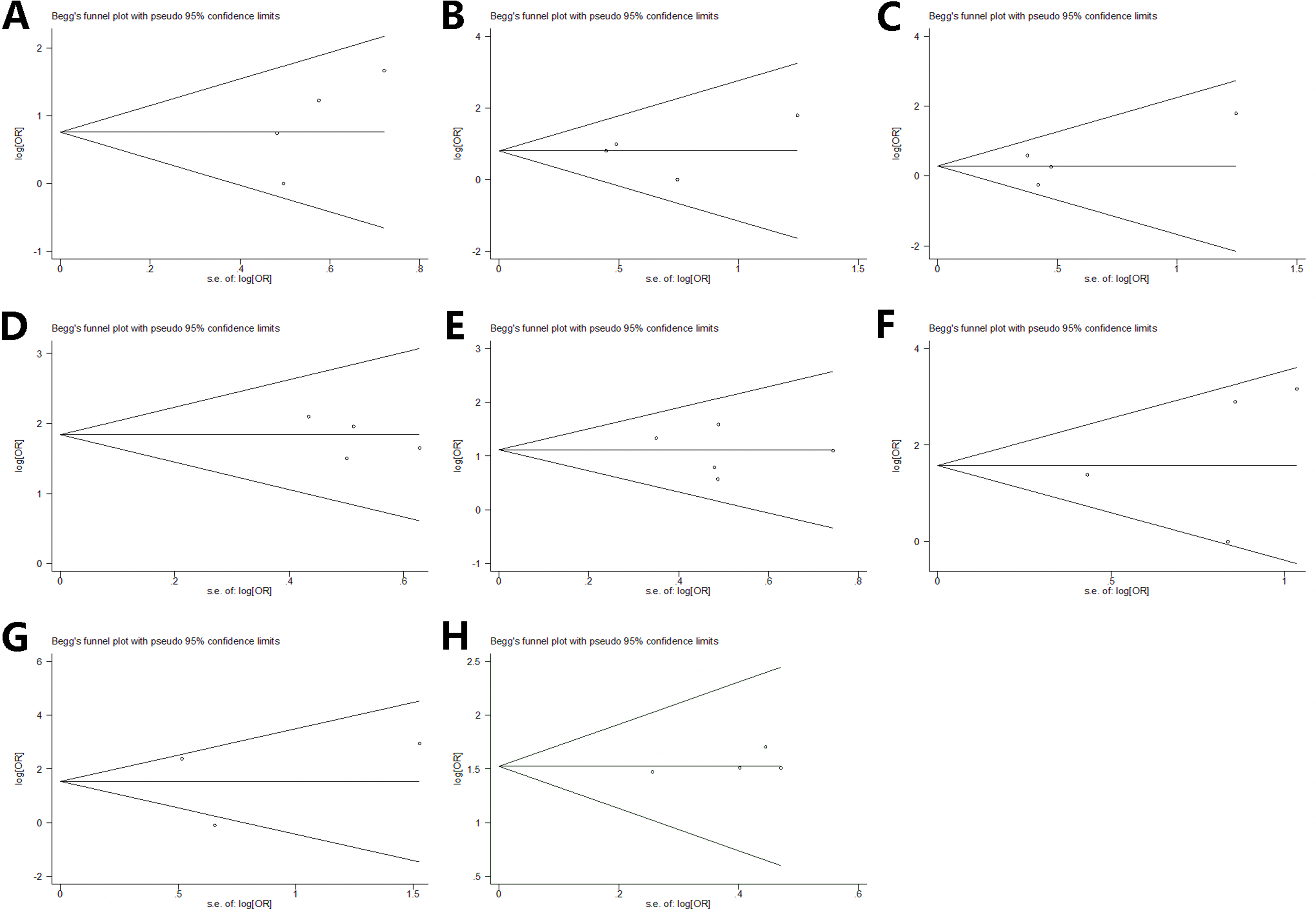  Figure 11. Publication bias of included studies. | | Result |
| Additional analysis | | 23 | | No additional analysis was carried out. | |  |
| **DISCUSSION** | | | | | |  |
| Summary of evidence | | 24 | | Our Study included 8 studies and 550 newborns with AIS. The pooled analysis indicates that the associations were found for AIS: preeclampsia (OR 2.14; 95% CI, 1.25 to 3.66), ventouse delivery (OR 2.23; 95% CI, 1.26 to 3.97), fetal heart rate abnormalities (OR 6.30; 95% CI, 3.84 to 10.34), reduced fetal movement (OR 5.35; 95% CI, 2.17 to 13.23), meconium-stained liquor (OR 3.05; 95% CI, 2.02 to 4.60), low Apgar score (OR 5.77; 95% CI, 1.66 to 20.04) and resuscitation at birth (OR 4.59; 95% CI, 3.23 to 6.52). | | Discussion |
| Limitations | | 25 | | This study has several limitations. Heterogeneity is a potential problem to affect meta-analysis results. For ‘low Apgar score’ and ‘arterial umbilical cord pH’, heterogeneity was discerned across some studies, we didn’t observed significant heterogeneity among other studies. For ‘reduced fetal movement’, only two studies (68 cases) were included, obviously the result of study included much more patients was more convincing.  Besides, many reasons, the retrospective nature of these studies and the different base-line characteristics, may affect the result. In our meta-analysis, most of the included studies were designed retrospectively. Given the retrospective nature of these studies, the observers made decisions based on medical record documentation. | | Discussi |
| Conclusions | | 26 | | There is a significant association between perinatal hypoxia factors and AIS. The result indicate that perinatal hypoxia maybe one of causes of AIS. Large scale prospective clinical studies are still warranted. | | Discussi |
| **FUNDING** | | | | | |  |
| Funding | 27 | All phases of this study were supported by:  The National Science Foundation of China (No.81330016 and 31171020 to Dezhi Mu; No. 81172174 and 81270724 to Yi Qu), the Major State Basic Research Development Program (2013CB967404), the Grants from Ministry of Education of China (313037, 20110181130002), the Grant from State Commission of Science Technology of China (2012BAI04B04), the Grants from Science and Technology Bureau of Sichuan province (2010SZ0280, 2011JTD0005). | | | |  |

*From:*  Moher D, Liberati A, Tetzlaff J, Altman DG, The PRISMA Group (2009). Preferred Reporting Items for Systematic Reviews and Meta-Analyses: The PRISMA Statement. PLoS Med 6(6): e1000097. doi:10.1371/journal.pmed1000097

For more information, visit: **www.prisma-statement.org**.

Page 2 of 2
